# Effects of high fat diet, ovariectomy, and physical activity on leptin receptor expression in rat brain and white fat tissue

**DOI:** 10.3325/cmj.2014.55.228

**Published:** 2014-06

**Authors:** Senka Blažetić, Irena Labak, Barbara Viljetić, Marta Balog, Sandor G. Vari, Zora Krivošíková, Martin Gajdoš, Patrícia Kramárová, Anton Kebis, Rosemary Vuković, Livia Puljak, Elizabeta Has-Schön, Marija Heffer

**Affiliations:** 1 Department of Biology, University of Osijek, Osijek, Croatia; 2 Department of Chemistry and Biochemistry, Faculty of Medicine, University of Osijek, Osijek, Croatia; 3 Department of Medical Biology and Genetics, Faculty of Medicine, University of Osijek, Osijek, Croatia; 4 International Research and Innovation Management Program, Cedars-Sinai Medical Center, Los Angeles, CA, USA; 5 Department of Clinical and Experimental Pharmacotherapy, Medical Faculty, Slovak Medical University, Bratislava, Slovakia; 6 Faculty of Public Health, Slovak Medical University, Bratislava, Slovakia; 7 Department of Histology and Embryology, School of Medicine, University of Split, Split, Croatia

## Abstract

**Aim:**

To evaluate in a rat animal model whether ovariectomy, high fat diet (HFD), and physical activity in the form of running affect leptin receptor (Ob-R) distribution in the brain and white fat tissue compared to sham (Sh) surgery, standard diet (StD), and sedentary conditions.

**Methods:**

The study included 48 female laboratory Wistar rats (4 weeks old). Following eight weeks of feeding with standard or HFD, rats were subjected to either OVX or Sh surgery. After surgery, all animals continued StD or HFD for the next 10 weeks. During these 10 weeks, ovariectomy and Sh groups were subjected to physical activity or sedentary conditions. Free-floating immunohistochemistry and Western blot methods were carried out to detect Ob-R in the brain and adipose tissue.

**Results:**

StD-ovariectomy-sedentary group had a greater number of Ob-R positive neurons in lateral hypothalamic nuclei than StD-Sh-sedentary group. There was no difference in Ob-R positive neurons in arcuatus nuclei between all groups. Ob-R distribution in the barrel cortex was higher in HFD group than in StD group. Ob-R presence in perirenal and subcutaneous fat was decreased in StD-ovariectomy group.

**Conclusion:**

HFD and ovariectomy increased Ob-R distribution in lateral hypothalamic nuclei, but there was no effect on arcuatus nuclei. Our results are first to suggest that HFD, ovariectomy, and physical activity affect Ob-R distribution in the barrel cortex, which might be correlated with the role of Ob-R in election of food in rats.

Obesity is one of the leading health issues worldwide, associated with an increased risk of morbidity and mortality (1). In 1997, the World Health Organization (WHO) formally recognized obesity as a global epidemic (2). Increase in body fat stores and obesity is caused by an imbalance between energy intake and energy expenditure (3,4). Since childhood obesity is a predictor of an increased death rate, the “obesity epidemic” may reverse the current declining rate of death from cardiovascular diseases (5). Factors that contribute to obesity can be environmental (6), social (7), behavioral (8), psychological (9), and genetic (10,11).

Women generally have more body fat than men (12). Nevertheless similar odds ratios were recorded in women and men for the association of abdominal obesity with acute myocardial infarction (13). Weight gain is common after menopause, indicating an association between hormones and fat stores (14). A large scale observational study found that both the body mass index and the level of physical activity were independent predictors of mortality and that a higher level of physical activity did not eliminate the risk associated with adiposity. At the same time, women who were both lean and physically active had the lowest mortality (15). In animal studies menopause can be induced by ovariectomy (OVX) (16).

Obesity can also be called a disorder of appetite and it is controlled by complex homeostatic mechanisms involving the hypothalamus and brainstem (17). Many gut peptides like cholecystokinin, ghrelin, glucagon-like peptide-1 (GLP-1), and -2 and peptide YY (PYY) act on the brain to control eating behavior (18). There are two different system for controlling feeding behavior: short-term and long-term (19). Short-term regulation involves neural signals from the GI tract and its hormones, like insulin, glucagon, and ghrelin (20). A hormone that functions mainly within long-term regulation is leptin (16 kD), a hormonal product of the obesity (*ob*) gen, primarily secreted by adipocytes (21) and released in the brain. It generates a feeling of satisfaction and acts like an appetite-suppressing agent. Circulating leptin levels are lower in ovariectomized rats (22).

Food intake is regulated via neural circuits located in the hypothalamus (23). Leptin acts via its leptin or Ob receptors (Ob-R) and is primarily expressed in hypothalamic neurons (19) especially in arcuate, ventromedial, and dorsomedial nuclei (24). Leptin is transported across the blood-brain barrier (BBB) by a saturable transporter (25). Ob-R is also detected in nonhypothalamic areas in the mice and in human brain neocortex, cerebellum, entorinal cortex, amygdale, and rostral medulla (26). Adipocytes, endothelial cells, and macrophages also have leptin receptor at its surface, which suggests autocrine and paracrine action for leptin in human adipose tissue (27). Association between the expression of Ob-R in target tissues and physiological and hormonal controlled processes is still unclear. Leptin receptors mRNA is found in each of the major components of the CNS “feeding” circuitry – the brainstem, hypothalamus, and is distributed reward centers (Allan brain) (28). Therefore, the aim of the current study was to evaluate whether HFD affects Ob-R distribution compared with StD specifically in the barrel field and piriform cortex compared to standard feeding centers in the hypothalamus. We supposed that the combination of OVX and HFD is interesting for further research on selected brain regions, which might be alleviated by physical activity. We also supposed that changes in Ob-R level in white fat tissue would correlate with the changes in brain regions.

## Material and methods

### Experimental animal model

**Figure 1:**
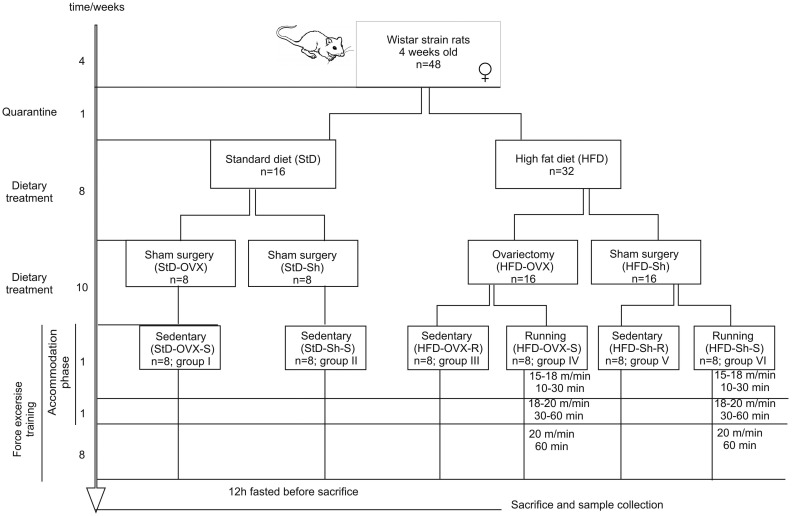
Flowchart of the experiment procedure.

The study was designed at the Department of Clinical and Experimental Pharmacotherapy, Medical Faculty, Slovak Medical University in Bratislava and all animal experimental procedures were conducted in animal husbandry in compliance with the standard operating procedures of the Department of Toxicology of the Slovak Medical University and the European Convention for the Protection of Vertebrate Animals used for Experimental and other Scientific Purposes. The experiment was designed in accordance with the current legislation on the use of experimental animal in Slovakia. The proposal was approved by the Ethics Committee for Animal Experiment of the Slovak Medical University and by the State Veterinary and Food Authority of the Slovak Republic. The study was conducted between October 2012 till April 2013 and included 48 female laboratory rats (Wistar strain, 4 weeks old, body weight 130-150 g), purchased from Charles River Wiga GmbH (Sulzfeld, Germany). The animals were housed in an air-conditioned room (with relative humidity 55 ± 5% and temperature 20-24°C) under a 12-hour light/dark cycle and *ad libitum* access to diet and water. After 7 days of quarantine, acclimatized rats were randomly divided into two dietary groups: a standard diet group (StD, 16 rats) (M3, Bonagro s.r.o., Czech Republic) and a high fat diet group (HFD, 32 rats) (D12451 /I/ mod. 45 kJ% fat, ssniff Spetzialdiätten GmbH, Soest, Germany). Subsequently, HFD rats were divided into running or sedentary group. Therefore, the rats were divided into six different groups: standard diet-ovariectomy-sedentary (StD-OVX-S), standard diet-sham-sedentary (StD-Sh-S), high fat diet-ovariectomy-running (HFD-OVX-R), high fat diet-ovariectomy-sedentary (HFD-OVX-S), high fat diet-sham-running (HFD-Sh-R), and high fat diet-sham-sedentary (HFD-Sh-S) (Figure 1).

### Ovariectomy

Following eight weeks of dietary treatment, rats from both dietary groups were subjected to either OVX (8 rats from StD and 16 rats from HFD) or Sh surgery (8 rats from StD and 16 rats from HFD). Rats were anesthetized with an intra-peritoneal injection of 10% ketamine (Narketan, Vetoquinol S.A., Lure Cedex, France) and 2% xylazine (Xylariem, Ecuphar N.V., Oostkamp, Belgium) at a ratio of 3:2 and a dose of 0.13 mL/100 g body weight. In 24 rats (8 from StD group, 16 from HFD group), bilateral OVX was performed using a single dorso-lateral approach (29,30). The remaining 24 rats from both dietary groups were subjected to Sh. After OVX, all rats continued with StD or HFD for another 10 weeks.

### Physical activity

Rats were divided into two groups based on participation in physical activity – exercise (running) and sedentary group. Eight rats from both HFD-Sh and HFD-OVX group were concurrently subjected to force exercise training (running) on a 4-channel treadmill (Harvard Apparatus, Holliston, MA, USA), whereas rats in the sedentary groups were placed on the turned-off treadmill for the same period. The exercise consisted of a 2-week accommodation phase with increasing exercise intensity (first week: 15-18 m/min for 10-30 minutes, second week: 18-20 m/min for 30-60 minutes), followed by an 8-week constant training period (20 m/min for 60 minutes). Before each training session (5 times a week), all running rats had a 5-minute warm-up phase with slowly increasing speed.

### Animal samples collection

Rats fasted for 12 hours before sacrifice. After deep anesthesia, samples of perirenal and subcutaneous fat were fresh frozen in liquid nitrogen (-196°C) and stored at -80°C till analysis. The rat brains were removed and post fixed for 24 hours in 4% paraformaldehyde solution in 1xPBS (phosphate buffered saline). After fixation, brains were cryoprotected in 10% sucrose for 24 hours followed by 30% sucrose in 1xPBS overnight at 4°C. Finally, the brains were frozen in cold isopentane and stored at -80°C till analysis.

### Immunohistochemistry

Paraformaldehyde fixed and cryoprotected brain samples were cut in the coronal plane (35 µm) on cryostat (Leica CM-3050-S, Leica Microsystems Nussloch GmbH, Nussloch, Germany) and free-floating immunohistochemistry was carried out to determine the distribution of leptin receptor (Ob-R). Sections were pre-treated in 1% H_2_O_2_/1xPBS solution for 30 minutes to block endogenous peroxidase activity and then incubated in 1% BSA and 5% goat serum in 0.1 M PBS blocking solution for 2 hours, followed by incubation in the primary Ob-R antibody (diluted 1:30, H-300, sc-8325, Santa Cruz Biotechnology, Inc., Santa Cruz, CA, USA) overnight. Incubation in the secondary antibody, biotinylated anti-rabbit IgG (diluted 1:500, Santa Cruz Biotechnology, Inc.) lasted 2 hours. After washing in 1xPBS, sections were incubated in Vector Elite peroxidase kit (ABC) (Vector Laboratories, Burlingame, CA, USA) for 1 hour, washed several times in PBS and then incubated with peroxidase substrate kit (DAB) (Vector Laboratories). All steps were performed at +4°C without using detergents. Sections were then mounted on slides, air dried, and scanned with Nikon Super CoolScan 9000 ED. After this, sections were coverslipped with Vectamount (Vector Laboratories). Microscopic images were acquired using an Olympus D70 camera (Olympus Corporation, Center Valley, PA; USA) mounted on Zeiss Axioskop 2 MOT microscope (Goettingen, Germany). Multiple images were assembled in Adobe Photoshop CS5 (Adobe Systems Inc., San Jose, CA, USA) for uniform adjustment of contrast, intensity, and brightness. Ob-R positive neurons were quantified using ImageJ software (NIH, Bethesda, MD, USA) (31).

### Western blotting analysis

Fresh frozen samples of perirenal and subcutaneous adipose tissue were homogenized in 100 mM phosphate buffer, pH 7.4 at +4°C. Total protein concentration was determined using Bradford assay. Since there were 6 groups of animals, samples from each group were pulled together in concentration of 5 mg/mL each. To perform Western blot, BioRad Mini Protean 3 Western transblot system was used with Bis-Tris 4%-12% gradient gels following the manufacturer’s protocol for running and transfer onto nitrocellulose membranes (BioRad, Hercules, CA, USA). After transfer, membranes were blocked in blocking solution (5% milk, 0.1% Tween 20 in PBS) for 1 hour at room temperature followed by incubation in primary antibodies overnight or up to 2 nights and shaking at 4°C. Ob-R antibody (Santa Cruz Biotechnology, Inc.) used to detect Ob-R in perirenal and subcutaneous adipose tissue was diluted 1:250 in blocking solution and anti-glyceraldehide-3-phosphate dehydrogenase (GAPDH) (Millipore, Bedford, MA, USA) used as internal control was diluted 1:5000 in blocking solution. The blots were washed in washing solution (PBS with 0.1% Tween 20) for 5 minutes four times, and placed in blocking solution with secondary antibodies goat anti-mouse-HRP (Santa Cruz Biotechnology, Inc.) and goat anti-rabbit-HRP (Santa Cruz Biotechnology, Inc.) for 1 hour at room temperature. Blots were washed five times in washing solution and one time in PBS for 5 minutes, and developed with Immun-Star WesternC Kit (Bio Rad) on photographic film.

### Anthropometric and biochemical measurements

Body weight was recorded for three times: at the beginning, before OVX, and at the end of the study, before sacrifice. Food consumed for each week was recorded in grams for individual rats. This was done for the entire study. The amount of food was calculated in calories based on food metabolizable energy (StD = 13.71 MJ/kg; HFD = 19.1 MJ/kg). Total body fat and abdominal fat were analyzed by a densitometer Lunar Prodigy Advance with Encore 2011 software version 13.60 (Ge Medical Systems, Madison, WI, USA). Insulin concentrations were assayed by ELISA method and glucose was measured by a Beckman Autoanalyzer CX5 using reagents provided by the manufacturer (Ramcon A/S, Birkerod, Denmark).

### Statistical analysis

All statistical analyses were performed using One-Way Univariate ANOVA (F-test). When there were more than two groups for the independent variable, the Bonferroni or Mann-Whitney post-hoc tests were performed, depending on the type of data. Statistical significance level was set at *P* ≤ 0.05. Statistical tests were performed using statistical software package SPSS, version 13.0 (SPSS Inc., Chicago, IL, USA) (32).

## Results

Four different brain regions were analyzed: arcuate nucleus (Arc), lateral hypothalamic nuclei (LH), barrel cortex (S1BF), and piriform cortex (Pir). Anthropometric and biochemical parameters were also calculated. To identify obesity the following parameters were measured: body weight (g), total body fat (g), abdominal fat (g), caloric intake (kcal/w), insulin (ng/mL), and glucose (mol/L) concentration. As expected, body weight increased with HFD (*P* = 0.009) and OVX (*P* = 0.001), compared to StD and Sh surgery, respectively.

### Effect of high fat diet

**Figure 2:**
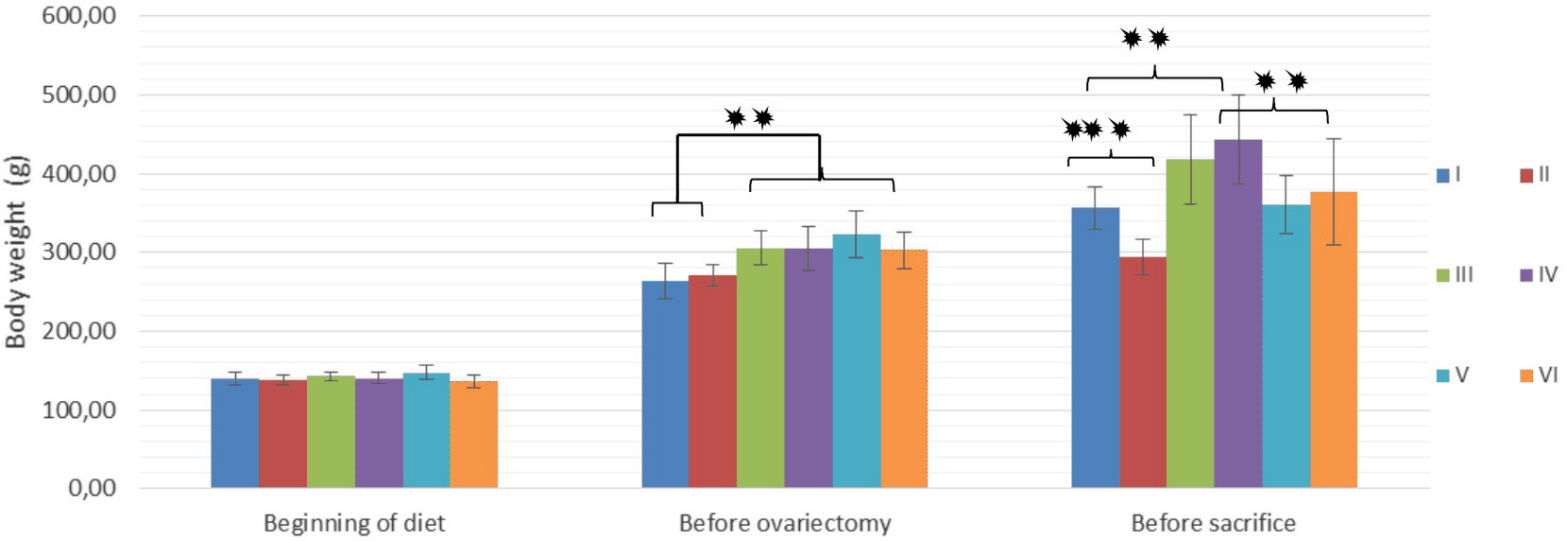
Mean body weight (g) per group in three different stages of experiment. Asterisks indicate a statistically significant difference between groups (**P ≤ 0.01; ***P ≤ 0.001) determined by Mann-Whitney test (I = StD-OVX-S; II = StD-Sh-S; III = HFD-OVX-R; IV = HFD-OVX-S; V = HFD-Sh-R; VI = HFD-Sh-S). Abbreviations: StD – standard diet; OVX – ovariectomy; S – sedentary; HFD – high fat diet; R – running; Sh – sham operated.

**Figure 3:**
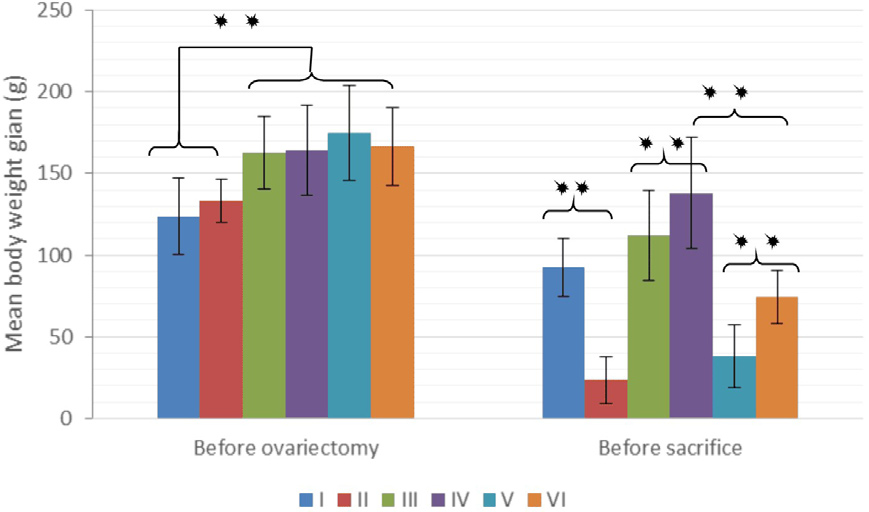
Mean body weight gain (g) per group before ovariectomy and before sacrifice. Asterisks indicate a statistically significant difference between groups (**P ≤ 0.01) determined by Mann-Whitney test (I = StD-OVX-S; II = StD-Sh-S; III = HFD-OVX-R; IV = HFD-OVX-S; V = HFD-Sh-R; VI = HFD-Sh-S). Abbreviations: StD – standard diet; OVX – ovariectomy; S – sedentary; HFD – high fat diet; R – running; Sh – sham operated.

**Figure 4:**
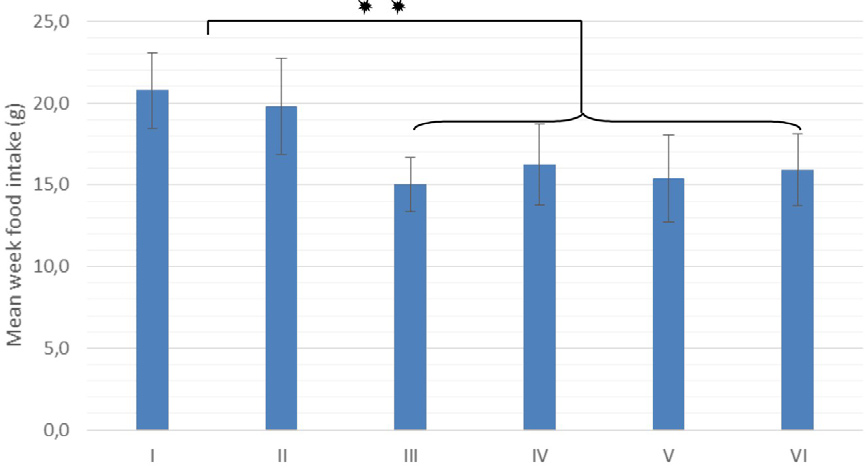
Mean week food intake (g) in the whole experiment. Asterisks indicate a statistically significant difference between groups (**P ≤ 0.01) determined by Mann-Whitney test (I = StD-OVX-S; II = StD-Sh-S; III = HFD-OVX-R; IV = HFD-OVX-S; V = HFD-Sh-R; VI = HFD-Sh-S). Abbreviations: StD – standard diet; OVX – ovariectomy; S – sedentary; HFD – high fat diet; R – running; Sh – sham operated.

**Figure 5:**
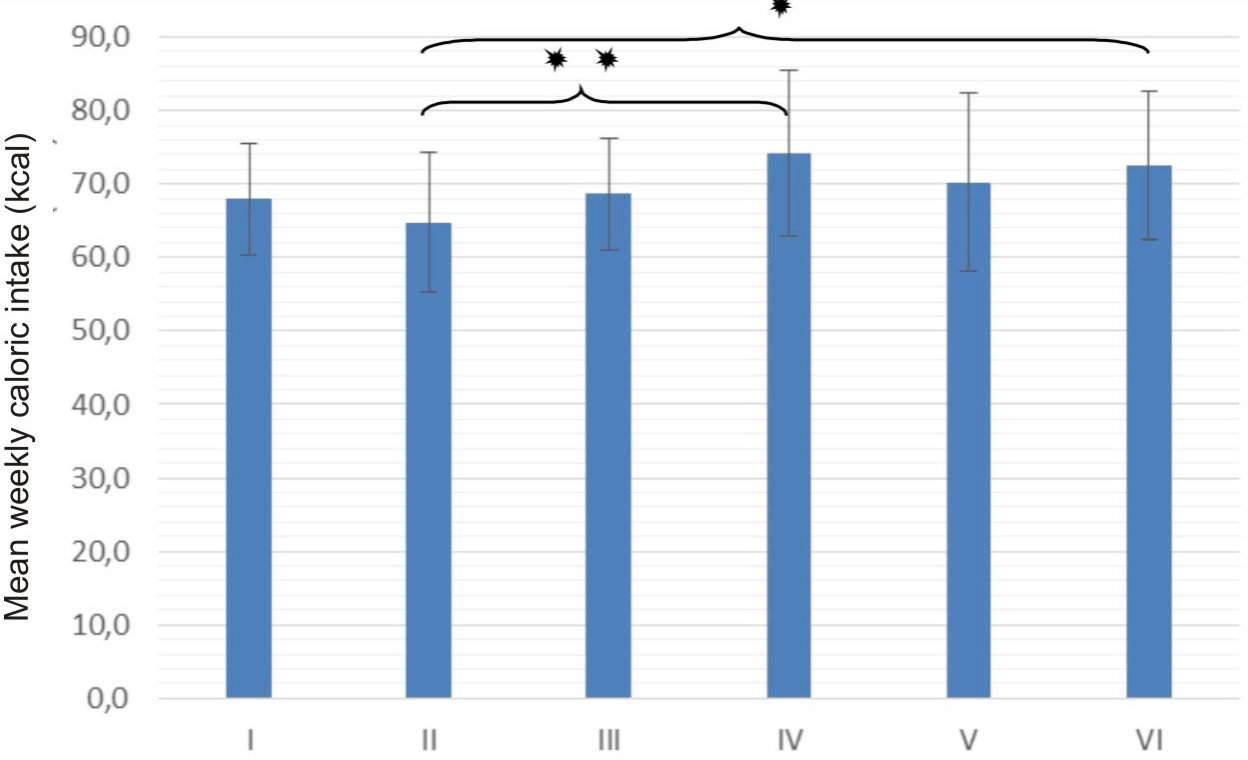
Mean week caloric intake (kcal) in the whole experiment. Asterisks indicate a statistically significant difference between groups (*P ≤ 0.05; ** *P* ≤ 0.01) determined by Mann-Whitney test (I = StD-OVX-S; II = StD-Sh-S; III = HFD-OVX-R; IV = HFD-OVX-S; V = HFD-Sh-R; VI = HFD-Sh-S). Abbreviations: StD – standard diet; OVX – ovariectomy; S – sedentary; HFD – high fat diet; R – running; Sh – sham operated.

**Figure 6:**
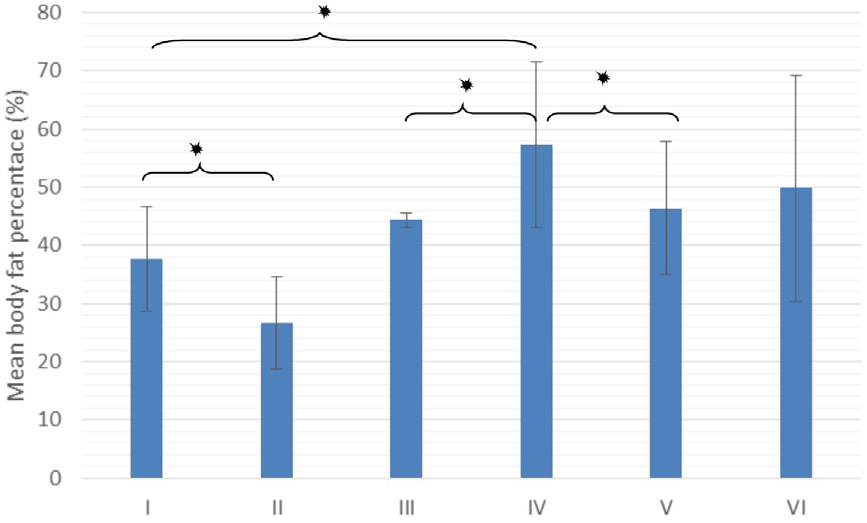
Mean body fat percentage (%) before sacrifice. Asterisks indicate a statistically significant difference between groups (*P ≤ 0.05) determined by Mann-Whitney test (I = StD-OVX-S; II = StD-Sh-S; III = HFD-OVX-R; IV = HFD-OVX-S; V = HFD-Sh-R; VI = HFD-Sh-S). Abbreviations: StD – standard diet; OVX – ovariectomy; S – sedentary; HFD – high fat diet; R – running; Sh – sham operated.

At the beginning of the study, all animals had similar weight (Figure 2). All animal groups gained weight in time, but weight gain was significantly higher in HFD than in StD (*P* = 0.007) groups (Figure 3). Mean weekly food intake was significantly higher in StD groups than HFD groups (*P* = 0.002) (Figure 4), but caloric intake was significantly lower in StD than in HFD groups (64.77 ± 9.53 kcal/w vs 74.14 ± 11.31 kcal/d, *P* = 0.007) (Figure 5). There was no significant difference in body fat percentage between HFD and StD groups after 8 weeks of dietary intervention (Figure 6). Also, there was no significant difference in insulin and glucose concentration based on the type of diet.

Immunohistochemical analyses of Ob-R positive neurons in hypothalamic nuclei, Arc, and LH did not show a significant difference between StD and HFD groups. Immunostaining in S1BF showed that StD groups had significantly fewer Ob-R positive neurons than HFD groups (*P* = 0.009). In Pir, there was no significant difference in Ob-R positive neuron distribution related to type of diet. Ob-R expression in perirenal and subcutaneous fat did not differ between StD and HFD rats.

### Effect of ovariectomy

**Figure 7:**
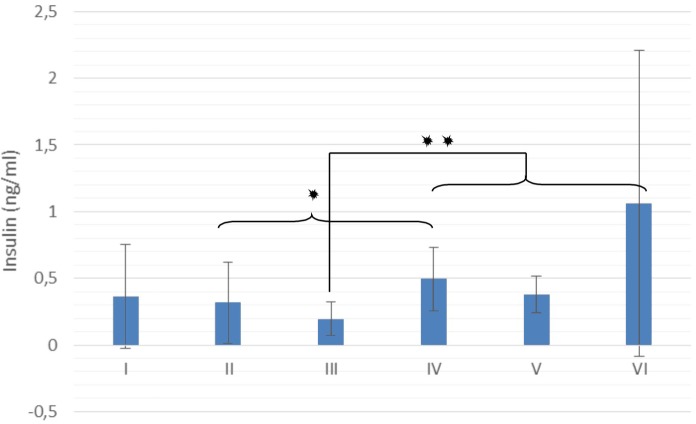
Insulin concentration (ng/mL). Asterisks indicate a statistically significant difference between groups (*P ≤ 0.05;**P ≤ 0.01) determined by Mann-Whitney test (I = StD-OVX-S; II = StD-Sh-S; III = HFD-OVX-R; IV = HFD-OVX-S; V = HFD-Sh-R; VI = HFD-Sh-S). Abbreviations: StD – standard diet; OVX – ovariectomy; S – sedentary; HFD – high fat diet; R – running; Sh – sham operated.

**Figure 8:**
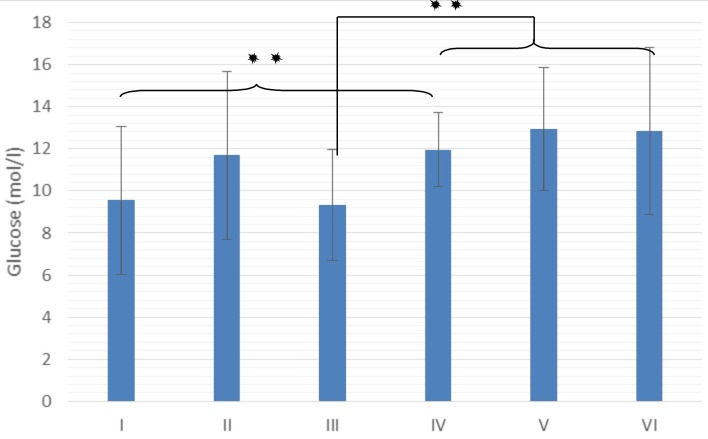
Glucose (mol/L). Asterisks indicate a statistically significant difference between groups (**P ≤ 0.01) determined by Mann-Whitney test (I = StD-OVX-S; II = StD-Sh-S; III = HFD-OVX-R; IV = HFD-OVX-S; V = HFD-Sh-R; VI = HFD-Sh-S). Abbreviations: StD – standard diet; OVX – ovariectomy; S – sedentary; HFD – high fat diet; R – running; Sh – sham operated.

OVX animals gained significantly more weight than Sh rats in both dietary groups. OVX caused significant body weight gain independent of the diet type, but OVX animals on HFD weighted significantly more than StD groups (*P* = 0.005) (Figure 3). OVX did not affect food and caloric intake in both dietary groups. OVX group on StD had significantly higher body fat mass than Sh group on StD (1.08 ± 0.33 vs 0.63 ± 0.16; *P* = 0.024). OVX animals on HFD (1.48 ± 0.26 vs 0.63 ± 0.16; *P* = 0.015) also had significantly higher body fat mass than OVX group on StD (Figure 6). Insulin concentration did not significantly differ between OVX and Sh animals on StD (Figure 7). The combination of HFD and OVX yielded significantly higher insulin concentration than the combination of StD and Sh (*P* = 0.03). Glucose concentration in HFD-OVX-S group was significantly higher than in StD-OVX-S group (*P* = 0.016) (Figure 8).

**Figure 9:**
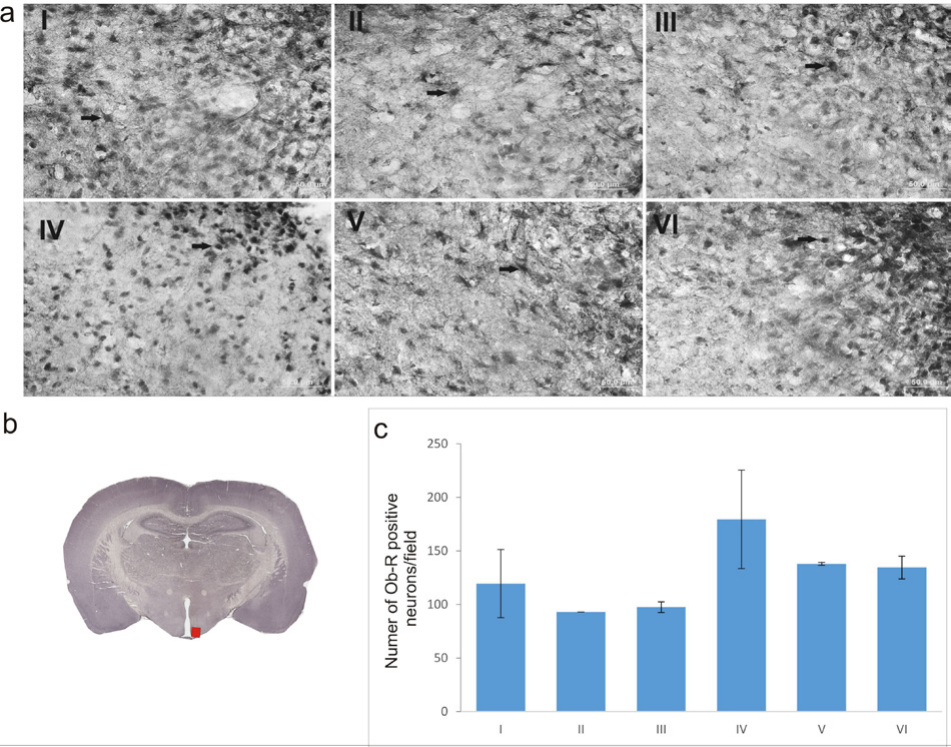
Immunostaining of Ob-R positive neurons (arrows) in arcuate nucleus; 20 × magnification (**A**). Coronal section of the rat brain. Square represents the analyzed nucleus (**B**). Data are presented as means ± standard error of the mean. There was no statistically significant differences between groups (I = StD-OVX-S; II = StD-Sh-S; III = HFD-OVX-R; IV = HFD-OVX-S; V = HFD-Sh-R; VI = HFD-Sh-S) (**C**). Abbreviations: StD – standard diet; OVX – ovariectomy; S – sedentary; HFD – high fat diet; R – running; Sh – sham operated.

**Figure 10:**
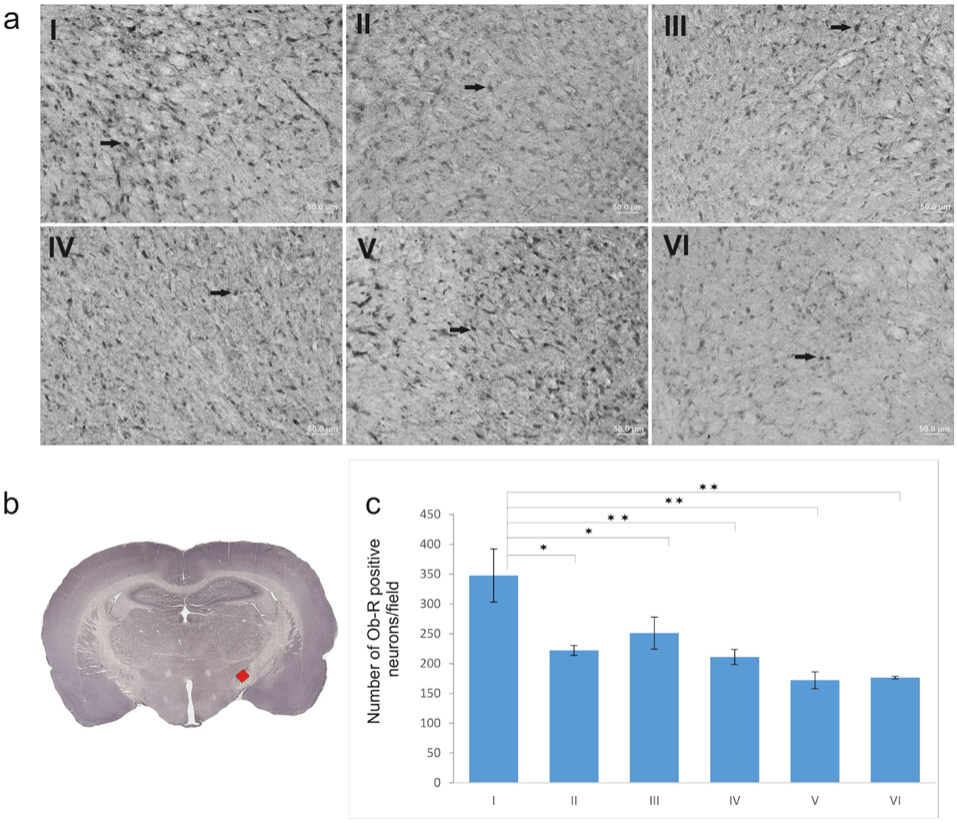
Immunostaining of Ob-R positive neurons (arrows) in lateral hypothalamic nuclei (LH); 20 × magnification (**A**). Coronal section of the rat brain. Square represents the analyzed nucleus (**B**). Data are presented as means ± standard error of the mean. Asterisks indicate a statistically significant difference between groups (*P ≤ 0.05; **P ≤ 0.01) determined by Tukey HSD test (I = StD-OVX-S; II = StD-Sh-S; III = HFD-OVX-R; IV = HFD-OVX-S; V = HFD-Sh-R; VI = HFD-Sh-S) (**C**). Abbreviations: StD – standard diet; OVX – ovariectomy; S – sedentary; HFD – high fat diet; R – running; Sh – sham operated.

**Figure 11:**
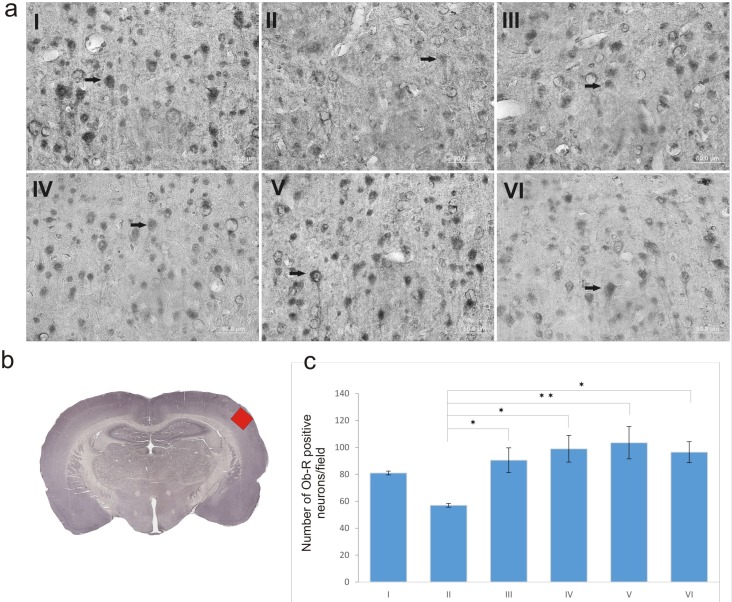
Immunostaining of Ob-R positive neurons (arrows) in the barrel cortex (S1BF); 20 × magnification (**A**). Coronal section of the rat brain. Square represents the analyzed region (**B**). Asterisks indicate a statistically significant difference between groups (*P ≤ 0.05;**P ≤ 0.01) determined by Tukey HSD test (I = StD-OVX-S; II = StD-Sh-S; III = HFD-OVX-R; IV = HFD-OVX-S; V = HFD-Sh-R; VI = HFD-Sh-S) (**C**). Abbreviations: StD – standard diet; OVX – ovariectomy; S – sedentary; HFD – high fat diet; R – running; Sh – sham operated.

**Figure 12:**
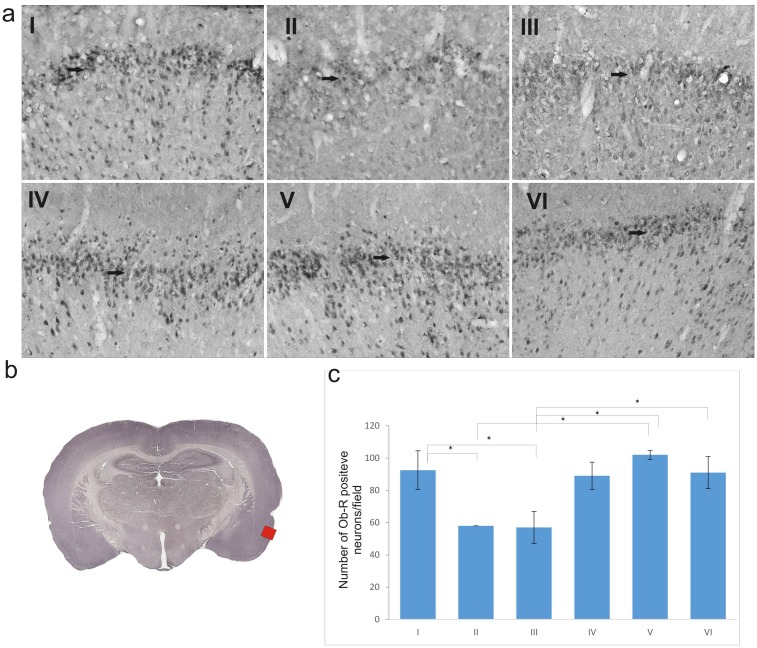
Immunostaining of Ob-R positive neurons (arrows) in the piriform cortex (Pir); 20 × magnification (**A**). Coronal section of the rat brain. Square represents the analyzed region (**B**). Data are presented as means ± standard error of the mean. Asterisks indicate a statistically significant difference between groups (*P ≤ 0.05) determined by Tukey HSD test (I = StD-OVX-S; II = StD-Sh-S; III = HFD-OVX-R; IV = HFD-OVX-S; V = HFD-Sh-R; VI = HFD-Sh-S) (c). Abbreviations: StD – standard diet; OVX – ovariectomy; S – sedentary; HFD – high fat diet; R – running; Sh – sham operated.

In Arc hypothalamic nuclei, there was no significant difference between OVX and Sh group in the number of Ob-R positive neurons (Figure 9). LH nuclei of StD-OVX-S group had a significantly greater number of Ob-R positive neurons than StD-Sh-S (*P* = 0.002) and HFD-OVX-S (*P* = 0.007) group, which indicates that OVX and HFD affect Ob-R distribution in the LH (Figure 10). In S1BF region there was no significant difference between OVX and Sh groups in the number of Ob-R positive neurons (Figure 11). Pir region of StD-OVX-S had significantly more Ob-R positive neurons than StD-Sh-S group (*P* = 0.006) (Figure 12).

**Figure 13:**
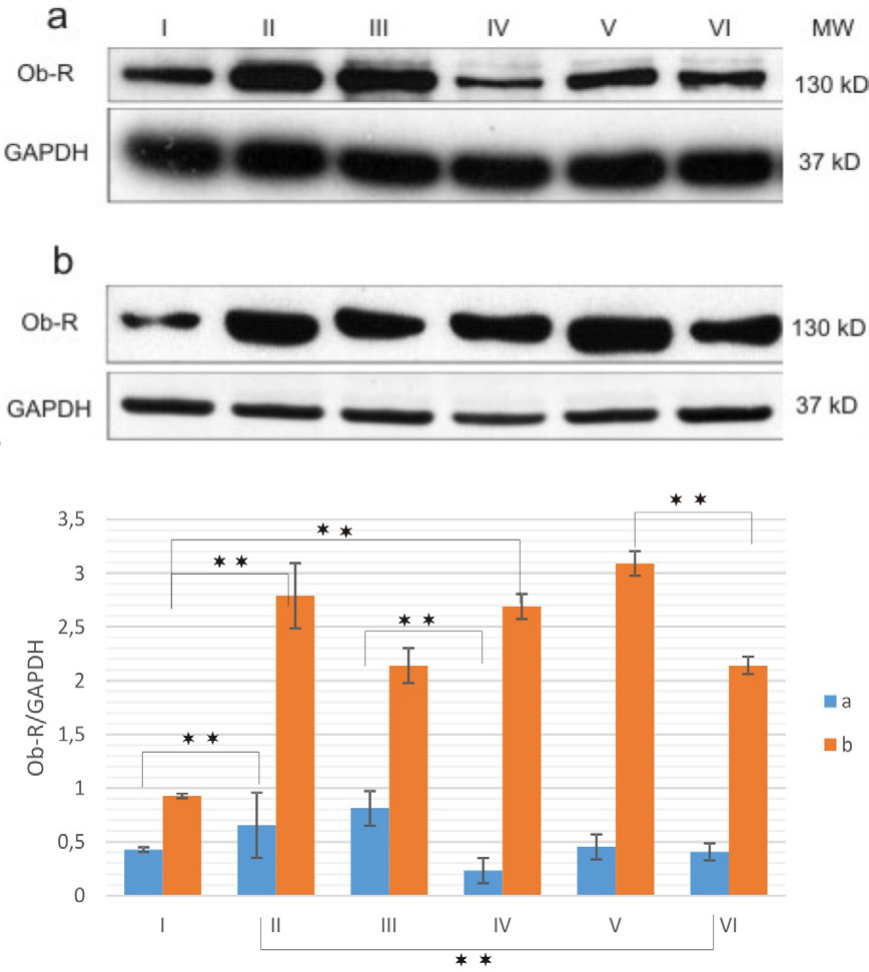
Evaluation of Ob-R (MW = 130kD) expression in rat perirenal (**A**) and subcutaneous (**B**) fat tissue by Western blot analysis. GAPDH (MW = 37kD) is used as an internal control. Asterisks indicate a statistically significant difference between groups (**P ≤ 0.01) determined by *t* test (I = StD-OVX-S; II = StD-Sh-S; III = HFD-OVX-R; IV = HFD-OVX-S; V = HFD-Sh-R; VI = HFD-Sh-S). Abbreviations: StD – standard diet; OVX – ovariectomy; S – sedentary; HFD – high fat diet; R – running; Sh – sham operated.

Ob-R expression was significantly decreased in the StD-OVX group in perirenal (*P* = 0.007) and subcutaneous fat (*P* = 0.010) compared to StD-Sh group. In subcutaneous fat, a significant increase in Ob-R expression was observed between StD-OVX-S and HFD-OVX-S group (Figure 13).

### Effect of physical activity in animals fed with high fat diet

HFD groups (OVX and Sh) gained more weight when they were not subjected to physical activity, and physical activity had less effect on OVX than Sh animals (Figure 3). HFD groups subjected to physical activity did not differ in caloric intake from StD groups not subjected to physical activity. Physical activity in combination with HFD and OVX significantly decreased body fat mass compared to HFD-OVX-sedentary group (*P* = 0.024) (Figure 6). Combination of HFD and physical activity in ovariectomized animal group yielded significantly lower insulin concentration compared to sedentary group (Figure 7). Glucose level was significantly lower in HFD-OVX group subjected to physical activity than in the sedentary group (*P* = 0.002) (Figure 8). Physical activity combined with HFD did not affect Ob-R distribution in the brain nuclei and brain regions. Physical activity increased Ob-R expression in perirenal fat of OVX-HFD group, while there was no change in subcutaneous fat. In Sh-HFD group physical activity also increased Ob-R expression in subcutaneous fat, while there was no change in perirenal fat (Figure 13).

## Discussion

This study showed that HFD, OVX, and physical activity affected Ob-R distribution in the brain and its expression in the perirenal and subcutaneous fat tissue. Immunohistochemical observations indicated that Ob-Rs were distributed throughout all parts of the brain.

The relationships between obesity and anthropometrical parameters in laboratory rodents are still poorly explored. Rats with free access to a HFD consume more calories and become obese compared to rats with free access to a diet containing the same constituents but less fat. Previous studies have demonstrated that fat accumulation is higher when more energy comes from dietary fat than from carbohydrate or protein (33). The difference in body weight and fat between the StD and HFD group can be ascribed to the relatively greater palatability of StD and/or to the difference in macronutrients and fat in the two diets (34).

### Ob-R distribution in the brain

In this study, Ob-R expression was analyzed in four different brain regions: Arc and LH, which are parts of the hypothalamus and barrel cortex and piriform cortex, which are nonhypothalamic regions. The two hypothalamic nuclei are important sites for the central regulation of food intake (35). Arc presents the “satiety center,” while LH presents the “feeding center” (29). In rats, whiskers are highly specialized sensory organs and a prime source of tactile and spatial information (30). This is why we analyzed changes in Ob-R expression in S1BF region, which is also called the “whisker barrels.” The piriform cortex processes information related to olfaction, mainly the perception and discrimination of smells, which is important in the feeding process. The results showed that there was no significant difference between the groups in Ob-R positive neurons in the Arc. A previous study also found no effect of diet on basal levels of total expression of Ob-R in the Arc. Much of the recent research has focused on the leptin-regulated circuitry of the hypothalamic Arc, although the majority of brain Ob-R-expressing neurons lie outside the Arc in other CNS regions known to modulate energy balance (29). The LH neurons project both within and outside the hypothalamus and modulate the activity of the parasympathetic and sympathetic nervous systems. In this study, OVX and HFD increased Ob-R presence in the LH, while physical activity combined with HFD did not affect the Ob-R distribution.

S1BF cortex had fewer Ob-R positive neurons in StD than HFD groups. In HFD group, there was no difference in Ob-R expression in S1BF based on OVX and physical activity. These results indicated possible role of Ob-R in election of food in rats, which is why in future studies rats should have an opportunity to choose between different types of food. This study is the first to report HFD, OVX, and physical activity effect on Ob-R positive neurons in the S1BF. Our results suggest that Ob-R modulates olfactory-mediated pre-ingestive behavior (30). Our study also showed that Pir region was most variable regarding Ob-R positive neurons. In StD group, OVX resulted in a higher number of Ob-R positive neurons compared to Sh group.

### Ob-R expression in subcutaneous and perirenal fat

Physical activity increased Ob-R presence in subcutaneous fat, while it had no effect in perirenal fat. This finding could be explained by the fact that apart from the elevation of energy expenditure, physical activity also increases resting energy needs (36). Ob-R expression was decreased in a StD-OVX-S group in perirenal and subcutaneous fat. A previous study showed that in the short-term (less than 10 weeks) Ob-R expression was higher in OVX than in Sh rats, while in the long-term (more than 10 weeks) it was lower in OVX than in Sh rats (37). This could mean that this system becomes overwhelmed and desensitized to circulating leptin. Based on these results, it is possible that OVX modulates Ob-R concentration in adipose fat.

In conclusion, modification of Ob-R expression in the brain and adipose tissue by HFD and physical activity indicates a connection between central and peripheral tissues in the regulation of body fat mass and weight gain. The combination of exercise and a low-fat diet might be helpful in obesity treatment (36). Also, this study provides more evidence that Ob-R is present in multiple brain regions involved in energy balance, including the brainstem, midbrain, and hypothalamus (38). Further studies of leptin are also likely to reveal additional links between nutritional state, physiology, and behavior. This study has several limitations: physical activity was not tested in StD groups, plasma levels of leptin and ghrelin were not analyzed, and there were no data about other peptides in the brain such as orexigenic neuropetide Y. Future studies should consider these issues to provide further insight into the studied mechanisms and variables.
